# Comparison between using hepatocellular carcinoma (HCC) risk scores and the HCC national guideline to identify high‐risk chronic hepatitis B patients for HCC surveillance in Thailand

**DOI:** 10.1002/jgh3.12753

**Published:** 2022-05-19

**Authors:** Chitchai Rattananukrom, Taya Kitiyakara

**Affiliations:** ^1^ Division of Gastroenterology and Hepatology, Department of Medicine, Faculty of Medicine, Srinagarind Hospital Khon Kaen University Khon Kaen Thailand; ^2^ Division of Gastroenterology and Hepatology, Department of Medicine, Faculty of Medicine, Ramathibodi Hospital Mahidol University Bangkok Thailand

**Keywords:** chronic hepatitis B, HCC surveillance, hepatocellular carcinoma, hepatocellular carcinoma risk score

## Abstract

**Background and Aim:**

Hepatocellular carcinoma (HCC) surveillance in hepatitis B virus (HBV) patients is currently based on age/sex/cirrhosis, uses ultrasound abdomen every 6–12 months, and is a resource burden. HCC risk scores have been developed to classify HCC risk for surveillance. The number of HBV patients needing surveillance when HCC risk scores are used may be different from the current recommendation with implications on the resources needed for HCC surveillance.

**Methods:**

HBV patients from the liver clinic were included and classified as non‐cirrhotic/cirrhotic and untreated/treated for analysis. Each subgroup was analyzed using REACH‐B, CU‐HCC, LSM‐HCC, GAG‐HCC, and mPAGE‐B risk scores as appropriate. The change in the number of patients needing HCC surveillance using the above risk scores was calculated.

**Results:**

Seven‐hundred and thirteen HBV patients were included, of whom 361 (50.6%) were male with mean age 55.43 years, and 76 (10.7%) had cirrhosis. In the untreated, non‐cirrhotic subgroup, the percentage change of patients needing HCC surveillance was −69.5, −58.9, −58.8, and −54.1% when GAG‐HCC, LSM‐HCC, CU‐HCC, and REACH‐B were used compared to traditional criteria, respectively. In the treated, non‐cirrhotic subgroup, the percentage change of patients needing HCC surveillance decreased by −80, −75.2, −75.2, and −2.8% when GAG‐HCC, CU‐HCC, REACH‐B, and mPAGE‐B were used, respectively. For the cirrhotic group, HCC risk scores did not make much difference.

**Conclusion:**

The use of HCC risk scores in non‐cirrhotic HBV patients reduced the number of patients needing surveillance greatly. HBV cirrhotic patients should have HCC surveillance without the need for risk score calculation. Patients with a family history of HCC should undergo surveillance until proven unnecessary in prospective trials.

## Graphical abstract

For non‐cirrhotic Hepatitis B patients, the number of patients requiring hepatocellular carcinoma (HCC) surveillance was much reduced when HCC risk scores were used, compared to the traditional age‐sex‐cirrhosis‐family history criteria for surveillance.

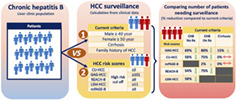

## Introduction

Chronic hepatitis B virus (HBV) is one of the commonest causes of liver cirrhosis and hepatocellular carcinoma (HCC). The World Health Organization (WHO) has estimated that there are more than 350 million HBV patients and approximately 1 million deaths per year from chronic HBV infection globally.^1^, ^2^ HBV is endemic in Thailand, and the incidence of HCC in Thailand is one of the highest in the world, with an age‐standardized incidence rate of 20–22/100 000. Similar high incidence rates are seen in Korea (14–20/100 000), China (13–18/100 000), and Japan (14/100 000). In contrast, lower rates are seen in Europe and the United States (6–9/100 000) and in India (3/100 000).^3^, ^4^

HCC surveillance is an important tool for early HCC detection, early treatment, and better outcomes. HCC surveillance is thought to be cost effective if the HCC incidence is more than 0.2% per year, or around 1% in 5 years.^5^, ^6^, ^7^

The method for HCC surveillance recommended by guidelines has traditionally been the use of ultrasound with or without an alpha‐fetoprotein (AFP) level check every 6 months. For ultrasound, the sensitivity is reported to be 58–89% and specificity >90% in HCC surveillance.^8^ A meta‐analysis showed that ultrasound had benefit for early HCC detection with a sensitivity of up to 94%.^9^ The obvious benefits of ultrasound are that it is a noninvasive test that is generally available and has the lowest cost compared to other imaging techniques, making it suitable for surveillance.^5^ Nevertheless, there is a resource burden associated with HCC surveillance. Surveillance requires the availability of ultrasound machines, and there are associated costs for training, manpower, and performing the procedures. This may be an important issue, particularly in HBV‐endemic countries where a significant proportion of the population will be within the surveillance criteria at some point during their lives. Many of these countries are also low‐ or middle‐income countries with limited resources to spare.

The Thailand Guideline for Management of Hepatocellular Carcinoma 2019 (TGMHC 2019 hereafter) recommend HCC surveillance with ultrasound with or without AFP every 6–12 months in high‐risk chronic hepatitis B patients with the following criteria^10^:

1. Males aged >40 years
2. Females aged >50 years
3. Cirrhosis with a Child–Pugh class A or B, or C if considering liver transplantation
4. A family history of HCC

As can be seen from the above criteria, all patients with hepatitis B will fall within the surveillance criteria at some point when they are old enough.

The Thai recommendation is similar to the Asia‐Pacific Clinical Practice Guidelines,^11^ which are also based on age/sex/cirrhotic status of HBV patients, the only difference being that the Thai guidelines accept a longer interval between ultrasound scans because of the real resource limitations for access to ultrasound scans in the country. In such a resource‐limited setting, a reduction in the number of needless ultrasound scans may open up appointment slots for patients who strongly need surveillance to have scans every 6 months.

More recently, HCC risk scores have been developed to help decide who should undergo HCC surveillance. These HCC risk scores have been developed to predict the probability of HCC development by analyzing many factors using large populations of HBV patients who were followed up for many years.

The current EASL Clinical Practice Guidelines: Management of Hepatocellular Carcinoma 2018 recommends HCC surveillance using the PAGE‐B score yearly.^5^ The PAGE‐B score was developed using a Caucasian population to classify the risk of HCC development in chronic hepatitis B patients. High‐risk patients should have an ultrasound every 6 months for HCC surveillance, while low‐risk chronic hepatitis B patients do not need to have a surveillance ultrasound but should be re‐assessed with the PAGE‐B score yearly.^12^

In the East, HCC risk scores for chronic hepatitis B patients have been developed in Hong Kong, Taiwan, and Korea. Each score was composed from many factors such as age, sex, cirrhosis, and HBV load. The scores can be separated in two groups: those to be used with naïve or untreated patients, and those to be used with treated patients.

For use in untreated patients, HCC risk scores include REACH‐B,^13^ CU‐HCC,^14^ GAG‐HCC,^15^ and LSM‐HCC.^16^ A summary of these risk scores is shown in Table A1, Appendix. For use in treated patients, HCC risk scores include Modified PAGE‐B,^17^ GAG‐HCC, CU‐HCC, and REACH‐B. A summary of these risk scores is shown in Table A2, Appendix.

Although there is no risk score specific for the Thai population, many of the risk scores developed in the East might be applicable for this population because of the genetic similarities of the population and similarities of the prevalent HBV genotypes. Many of the risk scores used in this study have already been validated in countries outside where they were developed.

The use of HCC risk scores to identify high‐risk chronic hepatitis B patients may lead to an increase or decrease in the number of patients needing surveillance. The implication of this change in the number of patients needing surveillance is that the resources needed to provide the surveillance (ultrasound procedures) nationwide would also change. If fewer patients need ultrasound at any one time, then fewer machines and less manpower will be needed to deliver that surveillance as well. However, as far as we are aware, there have been no previous studies directly comparing the use of HCC risk scores and the current HCC surveillance recommendation in Thailand to calculate the change in the number of patients needing surveillance. In this study, we compared the use of the HCC risk scores with the current HCC surveillance guidelines on a population of HBV patients seen in the liver clinic in a tertiary hospital in Bangkok, Thailand, to show the degree of change in the number of patients needing surveillance in a real population.

### Objectives

The primary outcome was to show the difference of HBV patients needing HCC surveillance when HCC risk scores were used, compared with using the recommendation from TGMHC 2019.

## Methods

A cross‐sectional study design was used, and patients were recruited from the GI and Liver Clinic in Ramathibodi hospital, Bangkok, Thailand, from 1 October 2019 to 30 September 2020. All chronic hepatitis B patients aged >18 years were included. Exclusion criteria were patients diagnosed with HCC, post liver transplantation, cirrhosis Child–Pugh C not wait‐listed for liver transplantation, coexisting liver disease, and chronic alcohol drinking. Patients were identified by going through the patient list for each clinic. Data were obtained from patient history, medical notes, and computer records.

All patients were evaluated according to the standard management using current Thailand Practice Guideline for Chronic Hepatitis B 2015 and evaluated for HCC surveillance strictly following TGMHC 2019. Patients were seen and treated in the clinic by consultant hepatologists, by gastroenterologist trainees, or by general medical residents who were supervised by consultant hepatologists.

The patients were divided into two groups: non‐cirrhotic and cirrhotic. Each group was subsequently separated into an untreated subgroup (or treated with nucleos[t]ide analogs [NUCs] for <2 years) and a treated subgroup (on NUCs for >2 years). The untreated subgroup was analyzed with REACH‐B, LSM‐HCC, CU‐HCC, and GAG‐HCC risk scores, while the treated subgroup (on NUCs for more than 2 years) was analyzed by mPAGE‐B, REACH‐B, GAG‐HCC, and CU‐HCC risk scores. The risk scores were chosen for each subgroup according to whether there were treated patients in their original study population.

Cirrhosis was defined by (i) finding clinical evidence of cirrhosis such as spider nevi or ascites, (ii) radiological evidence of cirrhosis, such as nodularity of the liver or splenomegaly, or (iii) a score ≥11 kPa with noninvasive testing such as elastography (fibroscan). Definition for untreated and treated group was based on the study from Hong Kong^18^:

1. The untreated group included chronic hepatitis B patients with or without cirrhosis who were not receiving NUC therapy, or have been receiving NUC therapy for <2 years.
2. The treated group included chronic hepatitis B patients with or without cirrhosis who have been receiving NUC therapy for >2 years.

### Choosing HCC risk scores and their cut‐points to compare with the current recommendation

We chose validated Asian HCC risk scores with high area under the receiver operating characteristic curve (AUROC) for 5‐year HCC predictions. These risk scores have been validated in other Asian populations and were assumed to be most generalizable and suitable for clinical practice in Thailand. We selected the cut‐point for each HCC risk score where the HCC prediction was ≥1% in 5 years, which we thought was approximately equivalent to the current HCC surveillance guidelines' recommendation for surveillance in patients with HCC risk of >0.2% in 1 year. Thus, the patients were categorized for each risk score as follows (Table 1):

1. High risk of HCC development were in patients with HCC prediction ≥1% in 5 years.
2. Low risk of HCC development were in patients with HCC prediction <1% in 5 years.

**Table 1:** Cut‐point for each risk score to determine high‐risk hepatitis B virus patients needing surveillance

HCC‐risk scores	HCC risk ≥1% in 5 years (cut‐point of each score)
CU‐HCC	≥5
GAG‐HCC	≥101
REACH‐B	≥9
LSM‐HCC	≥11
mPAGE‐B	≥9

HCC, hepatocellular carcinoma.

**Figure 1:**
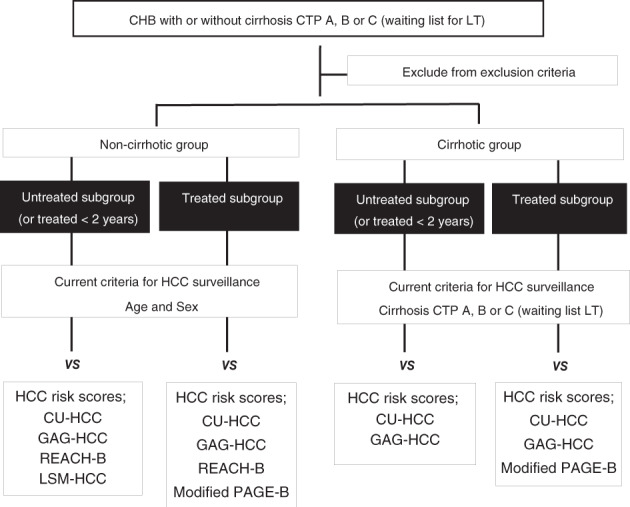
Study design showing categorization and hepatocellular carcinoma (HCC) risk scores used for each patient subgroup. CHB, chronic hepatitis B; LT, liver transplantion.

To determine which HCC risk scores should be used for each patient, HBV patients were classed into two groups: non‐cirrhotic and cirrhotic. Each group was subsequently divided into two subgroups: untreated (including those treated for <2 years), and treated (by definition, having been treated for ≥2 years). The untreated group was analyzed by CU‐HCC, GAG‐HCC, REACH‐B, and LSM‐HCC scores to identify high risk of HCC. The treated subgroup was analyzed by CU‐HCC, GAG‐HCC, REACH‐B, and mPAGE‐B scores to identify high‐risk patients for HCC (Fig. 1). The results of all subgroups were then compared with the results using the current HCC guidelines to determine the difference in the number of patients who should be recommended for HCC surveillance.

The study was approved by the Human Research Ethical Committee, Faculty of Medicine Ramathibodi Hospital, Mahidol university, COA. MURA2020/325 and Thai Clinical Trials Registry TCTR20200827002, and conducted in accordance with principles of the Declaration of Helsinki.

### Statistical analysis

We analyzed continuous variables expressed as mean ± SD or median (range), as appropriate. Categorical parameters were expressed as numbers and percentages. We compared the number of patients and the percentage of population change for HCC surveillance between using HCC risk scores and the current recommendation for HCC surveillance.

The change in the number and percentage of HBV patients needing HCC surveillance for a certain HCC risk score was determined by the method shown in the Table 2. A, B, C, D are the number of patients or percentages of the patient population falling into each category. A is the population (number or percentage) where both the risk score and the current guidelines do not recommend surveillance; B is where the risk score does not recommend surveillance but the current guidelines suggest that the patient should have surveillance; C is where risk score suggests the patient should have surveillance but the current guideline does not; and D is where the both the risk score and current HCC guidelines suggest surveillance is needed. The change in the number and percentage of the population need surveillance is (B + D) − (C + D) = B − C.

**Table 2:** Method of determining the change in number and percentages of patients needing hepatocellular carcinoma (HCC) surveillance for a certain risk score

		Current recommendation for HCC surveillance		
HCC risk score		No surveillance	Surveillance^†^	HCC surveillance % change
Risk‐score 1 example	Low risk = No surveillance	A	B	B–C
	High risk = Surveillance	C	D	

^†^ HCC surveillance: male >40 years of age, female >50 years; cirrhosis Child–Pugh class A, B, or C on waiting list for liver transplantation.

### Sample size calculation

$$n={\left[\frac{z_{1-\frac{\alpha }{2}}\sqrt{p_{01}+p_{10}}+z_{1-\beta }\sqrt{p_{01}+p_{10}-{\left(p_{01}-p_{10}\right)}^{2}}}{\Delta }\right]}^{2}.$$

We used the two‐dependent proportions test (two‐tailed test). We approximated for the proportion of pretreatment (*p*_10_) = 0.6 and the proportion of post‐treatment (*p*_01_) = 0.75 by alpha (*α*) = 0.05, *z* (0.975) = 1.959964 and beta (*β*) = 0.20, *z* (0.900) = 1.281552. The calculated sample size (*n*) was 627.

## Results

During the study period, data for 713 patients with chronic hepatitis B with or without cirrhosis were collected and analyzed from the liver clinic at Ramathibodi hospital. The demographic, virologic, and clinical characteristics of the patients are summarized in Table 3. The mean age was 55.43 ± 13.2 years, and 50.6% of the patients were male. A total of 76 patients (10.7%) had cirrhosis. For laboratory studies, HBeAg positive was 76 (10.7%), and HBV‐DNA was predominantly in low viral load level (median 19 IU/mL). The mean albumin, platelets, and fibroscan were 40.5 ± 3.5 g/L, 206 ± 62.2 × 10^9^/L, and 5.5 ± 2.6 kPa, respectively.

**Table 3:** Baseline characteristics (patients, *n* = 713)

Baseline characteristics	Total
Male (%)	361 (50.6)
Age, mean (SD), years	55.43 (13.2)
Untreated or treated for <2 years (%)	324 (45.4)
Treated for ≥2 years (%)	389 (54.6)
Cirrhosis (%)	76 (10.7)
Family history of HCC^†^ (%)	84 (16%)
HBeAg positive (%)	76 (10.7)
HBV‐DNA, median (IQR), IU/mL	19 (<10–532.5)
Total bilirubin, mean (SD), mg/dL	0.8 (0.4)
Albumin, Mean (SD), g/L	40.5 (3.5)
Platelets, Mean (SD), ×10^9^/L	206 (62.2)
Fibroscan^‡^, mean (SD), kPa	5.5 (2.6)

^†^ Data were collected from 523 patients.

^‡^ Only untreated group (or treated for 2 years).

HBV, hepatitis B virus; HCC, hepatocellular carcinoma, IQR, interquartile range.

We summarized the number and percentage of high‐risk HBV patients for each of the HCC risk scores, with their respective cut‐point for HCC risk ≥1% in 5 years (Table 4). The percentage of high‐risk patients for each HCC risk score was (in decreasing order) 81.1% for mPAGE‐B, 18% for CU‐HCC, 13.7% for LSM‐HCC, 10.5% for REACH‐B, and 9.4% for GAG‐HCC.

**Table 4:** The number and percentage of high risk patients (cut‐point hepatocellular carcinoma [HCC] risk ≥1% in 5 years) needing HCC surveillance in chronic hepatitis B with or without cirrhosis for each HCC risk score

HCC risk scores	HCC risk ≥1% in 5 years (cut‐point of score)	High risk (%)	Low risk (%)	Total (100%)
CU‐HCC	≥5	128 (18)	585 (82)	713
GAG‐HCC	≥101	67 (9.4)	646 (90.6)	713
REACH‐B	≥9	67 (10.5)	570 (89.5)	637^†^
LSM‐HCC	≥11	34 (13.7)	214 (86.3)	248^‡^
mPAGE‐B	≥9	314 (81.1)	73 (18.9)	387^§^
Current recommendation HCC surveillance	Male ≥ 40 years Female ≥ 50 years Cirrhosis Child–Pugh class A, B or C (waiting LT) Family history of HCC	555 (77.8)	158 (22.2)	713

^†^ REACH‐B score did not include cirrhotic patients.

^‡^ Only the untreated group (or treated for <2 years) with adequate data of fibroscan (75.6%).

^§^ Only the treated group ≥2 years with sufficient data.

**Table 5:** The results of the non‐cirrhotic, untreated subgroup (including treated for <2 years) (*n* = 318)

HCC risk score	HCC surveillance (high risk according to cut‐point^†^)	Current recommendation HCC surveillance		HCC surveillance % change
		Not needed (%)	Needed (%)	
GAG‐HCC	Not needed	94 (29.6)	221 (69.5)	−69.5
	Needed	0 (0)	3 (0.9)	
LSM‐HCC^‡^ *n* = 243	Not needed	69 (28.4)	145 (59.7)	−58.9
	Needed	2 (0.8)	27 (11.1)	
CU‐HCC	Not needed	84 (26.4)	197 (61.9)	−58.8
	Needed	10 (3.1)	27 (8.5)	
REACH‐B	Not needed	85 (26.7)	181 (56.9)	−54.1
	Needed	9 (2.8)	43 (13.5)	

^†^ High risk defined by the cut‐point HCC risk ≥1% in 5 years.

^‡^ Only patients with adequate data of fibroscan.

HCC, hepatocellular carcinoma.

**Table 6:** The results of the non‐cirrhotic treated subgroup (*n* = 319)

HCC risk score	HCC surveillance (high risk according to cut‐point^†^)	Current recommendation HCC surveillance		HCC surveillance % change
		Not needed (%)	Needed (%)	
GAG‐HCC	Not needed	64 (20.0)	255 (80.0)	−80.0
	Needed	0 (0)	0 (0)	
CU‐HCC	Not needed	59 (18.5)	245 (76.8)	−75.2
	Needed	5 (1.6)	10 (3.1)	
REACH‐B	Not needed	64 (20.0)	240 (75.2)	−75.2
	Needed	0 (0)	15 (4.7)	
mPAGE‐B *n* = 317^‡^	Not needed	58 (18.3)	15 (4.7)	−2.8
	Needed	6 (1.9)	238 (75.0)	

^†^ High risk is defined as cut‐point HCC risk ≥1% in 5 years.

^‡^ Two patients had inadequate data.

HCC, hepatocellular carcinoma.

### Comparison between HCC risk scores and the current recommendation from TGMHC 2019 to identify non‐cirrhotic HBV patients needing HCC surveillance

We compared the HCC risk scores and the current recommendation from the TGMHC 2019 to identify high‐risk HBV patients needing HCC surveillance. The results for the non‐cirrhotic patients are shown in Tables 5, 6. The 627 non‐cirrhotic patients were divided into two subgroups: 318 patients were in the untreated group (or treated <2 years), and 319 patients were in the treated group. In the non‐cirrhotic, untreated subgroup, we used four HCC risk scores: CU‐HCC, GAG‐HCC, REACH‐B, and LSM‐HCC, to compare with the current recommendation for HCC surveillance (Table 5). The results show that the number or percentage of patients needing HCC surveillance decreased dramatically when HCC risk scores were used. The percentage change of patients needing HCC surveillance was −69.5, −58.9, −58.8, and −54.1% when GAG‐HCC, LSM‐HCC, CU‐HCC, and REACH‐B were used, respectively.

In the non‐cirrhotic, treated subgroup, four HCC risk scores were used (CU‐HCC, GAG‐HCC, REACH‐B, and mPAGE‐B) to compare with the current recommendation for HCC surveillance (Table 6). The results show that the number and proportion of patients needing HCC surveillance decreased when the risk scores were used. The percentage of patients in the non‐cirrhotic treated group needing HCC surveillance decreased by −80, −75.2, −75.2, and −2.8% with GAG‐HCC, CU‐HCC, REACH‐B, and mPAGE‐B, respectively, compared with using the current national guidelines.

### Comparison between HCC risk scores and current recommendation from TGMHC 2019 for cirrhotic HBV patients

The 76 cirrhotic patients were also divided into two subgroups. Six patients were in the cirrhosis untreated subgroup (or treated for <2 years) and 70 patients were in the cirrhosis treated subgroup.

In the cirrhotic untreated subgroup, we used two HCC risk scores, CU‐HCC and GAG‐HCC, to compare with the current recommendation for HCC surveillance (Table 7). The percentage change of patients needing HCC surveillance was −16.7 and 0% (no change) when GAG‐HCC and CU‐HCC were used, respectively.

**Table 7:** The cirrhosis untreated group (including treated for <2 years) (*n* = 6)

HCC risk score	HCC surveillance (high risk^†^ according to from score)	Current recommendation HCC surveillance		HCC surveillance % change
		Not needed (%)	Needed (%)	
GAG‐HCC	Not needed	0 (0)	1 (16.7)	−16.7
	Needed	0 (0)	5 (83.3)	
CU‐HCC	Not needed	0 (0)	0 (0)	0
	Needed	0 (0)	6 (100)	

^†^ High risk defined as cut‐point HCC risk ≥1% in 5 years.

HCC, hepatocellular carcinoma.

In the cirrhosis treated subgroup, three HCC risk scores were used (CU‐HCC, GAG‐HCC, and mPAGE‐B) to compare with the current recommendation for HCC surveillance (Table 8). The percentage of patients needing HCC surveillance changed by −15.7, 0 (no change), and 0% (no change) when GAG‐HCC, CU‐HCC, and mPAGE‐B were used, respectively.

**Table 8:** The cirrhosis treated group (*n* = 70)

HCC risk score	HCC surveillance (high risk^†^ according to from score)	Current recommendation HCC surveillance		HCC surveillance % change
		Not needed (%)	Needed (%)	
GAG‐HCC	Not needed	0 (0)	11 (15.7)	−15.7
	Needed	0 (0)	59 (84.3)	
CU‐HCC	Not needed	0 (0)	0 (0)	0
	Needed	0 (0)	70 (100)	
mPAGE‐B	Not needed	0 (0)	0 (0)	0
	Needed	0 (0)	70 (100)	

^†^ High risk defined as cut‐point HCC risk ≥1% in 5 years.

HCC, hepatocellular carcinoma.

A sub‐analysis was performed to show the increase in the number of HBV patients needing surveillance when risk scores were used in the subgroup of patients who had low risk of HCC from the current recommendation from TGMHC 2019, who were below the screening age/sex criteria (male age below 40 years and female age below 50 years), who had no cirrhosis (*n* = 147). As shown in Tables 9, 10, the analysis was performed separately for the untreated (*n* = 83) and treated (*n* = 64) subgroups, respectively.

**Table 9:** Subgroup analysis for hepatitis B virus patients at low risk from the current guidelines: The untreated group (or treated group for <2 years) (*n* = 83)

HCC risk score	HCC risk ≥1% in 5 year (cut‐point of score)	High risk from risk score: HCC surveillance suggested, *n* (%)	Low risk from risk score: No surveillance needed, *n* (%)
CU‐HCC	≥5	10 (12%)	73 (88%)
GAG‐HCC	≥101	0 (0%)	83 (100%)
REACH‐B	≥9	7 (8.4%)	76 (91.6%)
LSM‐HCC *n* = 62^†^	≥11	1 (1.6%)	61 (98.4%)

^†^ Inadequate data = 21.

HCC, hepatocellular carcinoma.

**Table 10:** Subgroup analysis for hepatitis B virus patients at low risk from the current guidelines: The treated group for ≥2 years (*n* = 64)

HCC risk score	HCC risk ≥1% in 5 year (cut‐point of score)	High risk from score: HCC surveillance suggested, *n* (%)	Low risk from score: No surveillance needed, *n* (%)
CU‐HCC	≥5	5 (7.8%)	59 (92.2%)
GAG‐HCC	≥101	0 (0%)	64 (100%)
REACH‐B	≥9	0 (0%)	64 (100%)
mPAGE‐B	≥9	6 (9.4%)	58 (90.6%)

HCC, hepatocellular carcinoma.

In the untreated group (*n* = 83), the increase in the number of patients needing HCC surveillance using the risk scores were 10 (12%) in CU‐HCC, 7 (8.4%) in REACH‐B, 1 (1.6%) in LSM‐HCC, and 0 (0%) in GAG‐HCC. In the treated group (*n* = 64), the increase in the number of patients needing HCC surveillance were 6 (9.4%) in mPAGE‐B, 5 (7.8%) in CU‐HCC, 0 (0%) in REACH‐B, and 0 (0%) in GAG‐HCC, respectively.

Although HCC risk scores do not contain family history of HCC as a factor for calculating risk, it is a factor in the current recommendation from TGMHC 2019. We also found that during the year of study, one patient with a family history developed HCC. We therefore performed a sub‐analysis for patients with family history of HCC to show the difference in the number of HBV patients needing surveillance if family history of HCC were used additionally as a factor for surveillance on top of risk scores. We found that of the 719 patients whose notes were reviewed, 523 patients had documentation concerning family history of HCC. Of these 523 patients, 84 (16%) had a family history of HCC. Seventy‐seven had a family history in a total of 475 non‐cirrhotic patients, and 7 out of 48 cirrhotic patients had a family history of HCC. The results of the sub‐analysis for non‐cirrhotic patients and cirrhotic patients with family history of HCC are shown in Tables 11, 12, respectively.

**Table 11:** Sub‐group analysis for non‐cirrhotic hepatitis B virus patients with family history of hepatocellular carcinoma (HCC) (treated and untreated, *n* = 77)

HCC risk score	HCC surveillance (high risk according to cut‐point^†^)	Current recommendation HCC surveillance in those with family history	% difference in HCC surveillance out of total non‐cirrhotic patients (*n* = 475), *n* (%)
		Needed (%)	
GAG‐HCC	Not needed	76 (98.7)	76 (16%)
	Needed	1 (1.3)	
CU‐HCC	Not needed	71 (92.2)	71 (14.9%)
	Needed	6 (7.79)	
REACH‐B	Not needed	58 (75.3)	58 (12.2%)
	Needed	19 (24.7)	
LSM‐HCC Untreated *n* = 34	Not needed	30 (88.24)	—
	Needed	4 (11.76)	
mPAGE‐B Treated *n* = 37	Not needed	6 (16.21)	—
	Needed	31 (83.78)	

High risk or cut off is defined as ≥1% in 5 years.

**Table 12:** Sub‐group analysis for cirrhotic hepatitis B virus patients with family history of hepatocellular carcinoma (HCC) (*n* = 7)

HCC risk score	HCC surveillance (high risk according to cut‐point^†^)	Current recommendation HCC surveillance
		Needed (%)
GAG‐HCC	Not needed	1 (14.29)
	Needed	6 (85.71)
CU‐HCC	Not needed	0 (0)
	Needed	7 (100)
mPAGE‐B (*n* = 6)	Not needed	0 (0)
	Needed	6 (100)

High risk or cut off is defined as ≥1% in 5 years.

In the non‐cirrhotic, chronic hepatitis B patients with family history of HCC, in the untreated group (*n* = 40), the number of patients needing HCC surveillance was 16 (40%) in REACH‐B, 5 (12.5%) in CU‐HCC, 4 (11.76%) in LSM‐HCC, and 1 (2.5%) in GAG‐HCC. In the treated group (*n* = 64), the number of patients needing HCC surveillance was 31 (83.78%) in mPAGE‐B, 3 (0.08%) in REACH‐B, 1 (2.7%) in CU‐HCC, and 0 (0%) in GAG‐HCC (data not shown). Combined together, for non‐cirrhotic patients with a family history of HCC, the difference that family history of HCC makes between the current recommendations and the risk scores was 16% for GAG‐HCC, 14.9% for CU‐HCC, and 12.2% for REACH‐B risk scores, as shown in Table 11.

In cirrhotic patients with family history of HCC, three HCC risk scores were used (CU‐HCC, GAG‐HCC, and mPAGE‐B) to compare with the current recommendation for HCC surveillance (Table 12). The percentage of patients needing HCC surveillance changed by −14.29, 0 (no change), and 0% (no change) when GAG‐HCC, CU‐HCC, and mPAGE‐B were used, respectively.

## Discussion

HBV infection is one of the commonest causes of liver cirrhosis and HCC. In Thailand, liver cancer is the most common cancer in men and seventh commonest cancer in women, according to hospital‐based cancer registry 2019 from National Cancer Institute 2019,^19^ and HBV patients account for more than 50% of HCC cases in Thailand.^20^, ^21^

Many patients with HCC present late—when symptoms occur and the cancer is in the late stages—with poor treatment outcomes. Early diagnosis of HCC improves outcomes as curative HCC treatment can be performed. HCC surveillance program in chronic hepatitis B patients is an important tool to identify HCC in the early stages.

However, HCC surveillance as recommended by TGMHC 2019 using ultrasound with or without AFP every 6–12 months is a resource burden in that country. Each ultrasound procedure needs an ultrasound machine and someone trained to use it and interpret the findings, and all this requires money. Data from the Thailand National Statistical Office show that the Thai population was 66.55 million in 2019 and those aged >40 years were 31.74 million approximately.^22^ The estimated HBV patients aged >45 years in Thailand was 1.8 million.^23^, ^24^ Therefore, theoretically, 1 800  000–3 600  000 ultrasound procedures would be needed for nationwide surveillance every year. Many of these procedures will be unnecessary and no HCC will be detected. A more accurate and cost‐effective method for selecting HBV patients for surveillance would therefore reduce the number of ultrasound procedures needed as well as the resource burden of HCC surveillance.

HCC risk scores have been developed to predict HCC development in HBV patients and classify HBV patients needing HCC surveillance more accurately. Consequently, we wanted to see whether the use of HCC risk scores in a real population of patients would reduce the number of HBV patients needing HCC surveillance when compared to the current national recommendations.

In this study, we compared the use of HCC risk scores with the current recommendation for HCC surveillance from TGMHC 2019 in HBV patients attending the liver clinic over 1 year. From previous studies, an HCC surveillance program is considered cost effective if the HCC incidence is more than 0.2% per year, or approximately 1% in 5 years^5^, ^6^, ^7^; this was the cut‐off we used in our study.

As some of the risk scores were developed from certain HBV populations, for example, from non‐cirrhotic patients or from treatment‐naïve patients, we categorized our own patients accordingly so that the HCC risk scores would not be used in HBV patients with characteristics they were not developed for. As a result, we compared the HCC risk scores and the current recommendation in four subgroups; non‐cirrhotic, untreated; non‐cirrhotic, treated; cirrhotic, untreated; and cirrhotic, treated.

For non‐cirrhotic patients, both the untreated and treated subgroups, the results showed that the number or percentage of patients needing HCC surveillance would decrease dramatically if HCC risk scores were used instead of the current national guidelines. A 50–70% reduction in the number of non‐cirrhotic, untreated patients needing HCC surveillance was seen when HCC risk scores were used. For the non‐cirrhotic, treated group, there was a wider variation in the reduction in the number of patients recommended to have HCC surveillance with the risk scores. Most risk scores (GAG‐HCC, CU‐HCC, REACH‐B) reduced the number of patients by 75–80%; however, the m‐PAGE risk‐score reduced the number by only 2.8%. The reason for this difference is that the m‐PAGE risk‐score was mainly calculated by using the age and sex, but not viral load, similar to the current guidelines for HCC surveillance (see Table A2, Appendix for the risk score parameters). By excluding the m‐PAGE risk score, the percentage change in the non‐cirrhotic, treated subgroup of patients needing HCC surveillance showed a greater reduction than the non‐cirrhotic, untreated subgroup. The likely reason for this is that HBV viral suppression with antiviral therapy is an important factor in decreasing the risk of HCC development in HBV patients.^18^ This is adjusted for in the other risk scores but not in the traditional/current national guideline recommendations, which only look at age and sex and presence of cirrhosis in the patient. As a result, there is a larger change in the treated subgroup of patients compared to the non‐cirrhotic, untreated subgroup.

For HBV‐related cirrhotic patients, the percentage needing HCC surveillance did not change very much when risk scores were used, compared with the current national HCC recommendation. From previous studies,^6^, ^25^, ^26^, ^27^, ^28^, ^29^ cirrhosis was found to be a major risk factor of HCC development. The incidence of HCC development in HBV‐related cirrhosis was 1.5–8% per year. Both the current guidelines and the risk scores took into account the high risk of HCC development associated with cirrhosis and, as a result, both generally recommend surveillance for patients with cirrhosis.

We also performed subgroup analyses for patients younger than the recommended age for surveillance in the current guidelines and for patients with family history of HCC to see whether there was much change in these groups when risk scores were used. In young non‐cirrhotic patients, the majority were not re‐classified as high risk and would not need surveillance. The change to surveillance ranged from 0 to 12% of these low‐risk patients in GAG‐HCC and CU‐HCC, respectively. For those with a family history of HCC, if the family history is to be used as a separate indication for surveillance, we found that an extra 12–16% of non‐cirrhotic patients would need surveillance on top of those recommended purely by the risk scores.

The strengths of our study are as follows: (i) This was the first study to directly compare HCC risk scores and the traditional/current HCC surveillance recommendation to identify high risk for HCC development in non‐selected chronic hepatitis B patients with or without cirrhosis. The HCC risk scores showed a significant decrease in the percentage of patients (50–80%) needing HCC surveillance, especially in non‐cirrhotic HBV patients. (ii) The patient population used for this comparison was a real patient population attending a general liver clinic in a tertiary hospital over 1 year. This allowed all the data in this study to be checked and collected in a real‐time database.

However, our study had several limitations. First, our study did not determine the long‐term accuracy of each HCC risk score used in the comparison for our local population. Accuracy of risk scores is generally determined prospectively, after a follow‐up of 5–10 years, and as our study was only over 1 year, it could not be used to demonstrate which risk score was more accurate in our patient population. Although the risk scores have been validated in other Asian populations, only one study in Thailand has reported the accuracy of risk scores.^30^ The reported AUROC for GAG‐HCC, REACH‐B, and CU‐HCC in that study was 0.80, 0.79, and 0.73, respectively. Further long‐term follow‐up of the patients in this study would help determine which risk score is more accurate and which risk score should be used in Thailand in the future. It should also be noted that the primary aim of this study was not to validate or compare the accuracy of the risk scores in our population but it was to demonstrate the amount of reduction of patients needing surveillance when risk scores are used. The reduction in the resources needed for surveillance in itself is an important issue in a resource‐limited country with a national health system, such as Thailand, and has not been reported previously. In addition, a reduction in the number of unnecessary ultrasound scans may improve access to ultrasound scan for those who are at high risk by opening appointment slots.

Another limitation was that there were data missing for some of our patients. Such data include elastography/fibroscan data. Fibroscan measurements are needed for HCC risk score (LSM‐HCC) calculation. Conversely, if the fibroscan score was inaccurate and misclassified the patient for cirrhosis, the patient may have ended up not having HCC surveillance when it should be done.

Another limitation was that HCC risk scores do not include the family history of HCC as a significant risk factor. From history taking and medical notes, we found that approximately 16% of our patients had a family history of HCC. The limitations described above are demonstrated in the fact that, for the two patients in whom new HCC developed during the year of study, one had a strong family history of HCC (he was also >40 years old and was screened and detected by the current screening recommendations) and the other patient was misclassified by fibroscan as non‐cirrhotic (cirrhosis was confirmed at hepatectomy) but was screened because of her age. If risk scores had been used instead of the current recommendation, and if the inaccurate fibroscan value was used in the risk score calculation rather than the true cirrhotic status of her liver, then both would have been classified as low risk for HCC and “missed” by the risk scores. From this experience, we suggest that patients with a family history of HCC should have HCC surveillance as an independent factor if HCC risk scores are used.

Lastly, our study used the patient population from a liver clinic from a tertiary hospital. This population may be different from the HBV patient population in general practice. The degree of reduction may, therefore, also be different. Also, it is unclear whether risk scores would be used consistently in general practice where many of the HBV patients are followed up, and this would need to be tested in another study. One may also question whether a national registry of patients, if available, would be preferable to using the patient population from a liver clinic to calculate the benefit of using risk scores. Unfortunately, there are no accurate national registries of HBV patients or HCC patients at present in Thailand. Reimbursement data for hospital admissions for HCC are available from the National Health Service Office (NHSO), which is the national healthcare provider covering approximately 47 million out of 68 million people in Thailand. However, the coding for secondary diagnosis and the clinical data in this dataset are not accurate enough to determine how many patients with HCC have hepatitis B, how many have underlying cirrhosis, and whether patients would be in the low‐risk or high‐risk group, using the risk scores. In any case, it would be difficult to back‐model from the incidence of HCC in Thailand to calculate the reduction of surveillance using risk scores, as the number of HCC patients, or incidence in the general population, does not imply any particular ratio of low‐risk to high‐risk HBV patients in the population. As for the HBV data, the NHSO data is predominantly an inpatient reimbursement dataset, and the majority of HBV patients do not require inpatient treatment and thus the HBV data would not be particularly accurate or useful for this study.

One other point, despite the understanding that national screening/surveillance programs are predominantly assessed by their cost effectiveness, and that cancers will be missed even when a surveillance program is put in place, the question of missed cancers remains a clinically interesting one.

These missed cancers may result from patients falling outside the surveillance criteria due to their level of perceived risk (referred to from here on as unsurveilled‐HCC to differentiate from the other causes of missed HCC) or from a failure to detect the cancer at ultrasound or from appointment non‐attendance. It may be useful for policy planning to have an approximation of what percentage of HCCs would be missed because patients with HCC fall outside the surveillance criteria (unsurveilled‐HCC) when the traditional age–sex–cirrhosis criteria is used compared with risk scores, and their corresponding reduction in surveillance.

First, it should be noted that many countries have different stratification criteria and different intensities of surveillance for HCC, depending on the concern they have on the risk and burden of HCC in their respective country and their ability to afford the surveillance program. For example, the Japanese Society of Hepatology guidelines^31^ recommend a 3–6‐monthly ultrasound scan and an array of tumor markers for the very high‐risk and high‐risk patients, as well as additional contrast enhanced ultrasonography to detect the HCC. High‐risk groups include patients with chronic hepatitis B and chronic hepatitis C, and very high‐risk groups are patients with viral hepatitis and cirrhosis. A recent multi‐committee guideline from China^32^ included stratification using the REACH‐B criteria (cut‐off point of 12 for high risk) as well the traditional age–sex and family history parameters for HBV patients. In contrast, many poorer countries lacking strong healthcare infrastructure may not have surveillance for HCC. This implies that the traditional age–sex–cirrhosis surveillance criteria may be imperfect and some balance of cost effectiveness is used for each country. However, direct head‐to‐head comparisons of the benefit of different surveillance criteria have for the most part not been performed.

Data on unsurveilled HCC, particularly from patients falling outside the traditional age–sex–family history HBV surveillance criteria in study populations, are often unreported and were not referred to in the published national guidelines.^5^, ^6^, ^11^, ^31^, ^32^ Comparative trials such as the randomized cohort trial in China^33^ and meta‐analysis^34^ studying the relative benefit of HCC surveillance compared to non‐surveillance in cirrhotic patients did not mention this data on unsurveilled HCC.

A rough estimate of the proportion of unsurveilled‐HCC in Thailand from the traditional age–sex criteria may be obtained from historical government data. Reimbursement coding data from the three national healthcare funds (the NHSO, the Civil Service Healthcare Fund, and the Social Insurance Healthcare fund) in Thailand covering all citizens in Thailand, presented in abstract form,^35^ showed that in 2010, there were 15  290 patients admitted with HCC in the country (as defined by an ICD‐10 code C22.0). Of these, 834 were male and under the age of 40 years, and 686 were female and under the age of 50 years. Although not all these HCC patients would be HBV patients, hepatitis B is the predominant cause of HCC in Thailand, particularly in the younger age groups. Therefore, taken as an approximation, the percentage of HCC in the segment of population below the HBV surveillance criteria for age–sex was 9.94%. In the same dataset, there were 2683 patients coded as having a combination of HCC and HBV. This number was much lower than expected and is likely to be inaccurate as local registries have shown that HBV infection account for at least 70% of all HCC in Thailand.^36^ However, if we look within this group, there were 206 males and 158 females who were below the sex‐specific age criteria for surveillance for chronic HBV. As a proportion of this group, this amounted to 13.57%. Interestingly, using the sex‐specific age cut‐off, the proportion of missed HCC in females was higher than in males in this group (30.1 *vs* 9.6%). No data were available to determine how many of this subgroup of patients had family histories of HCC or cirrhosis and would have qualified for surveillance from these criteria independently.

In contrast, there have been some reports on the proportion of unsurveilled‐HCC from studies using HCC risk scores. The negative predictive values for the risk scores used in these studies vary from 95^15^ to 100%^18^ Most commonly, the negative predictive values fall around the 98^14^, ^15^ and 99%^18^ range. The AUROC in East Asian populations has been reported as 0.75–0.88.^13^, ^14^, ^15^, ^16^, ^17^ The sensitivities of the risk scores for classifying HCC correctly in high‐risk patients in untreated or treated HBV for <2 years have varied according to the studies. For REACH‐B, the sensitivity for classifying HCC correctly was 95–100%,^18^ for GAG‐HCC 67–68%,^14^ for CU‐HCC 69–86%,^15^ and for LSM‐HCC 92%.^16^

In a recent study comparing various HCC risk scores in a Thai population,^30^ the negative predictive values for every risk score analyzed were above 99%. However, the sensitivities of the risk scores were generally lower than previously described in the original study and validation studies, with REACH‐B having a sensitivity of 85%, CU‐HCC 35%, GAG‐HCC 25%, and mPAGE‐B 20%. Although this study was performed in a Thai population, there were also some differences to our population. Their population appeared to be at an earlier stage in HBV infection. The proportion of patients with cirrhosis was only 3% compared to our 10%, with a mean age of 41.3 years compared to our 55 years, and most of their patients were not on antivirals at entry into the study. It is possible that risk scores and even the traditional age–sex criteria for surveillance would be less sensitive in younger, non‐cirrhotic samples of the population, as the proportion of HCC developing in young, non‐cirrhotic patients falling outside the criteria would also be higher, even though the overall risk and rate of developing HCC per year in this subgroup are low, simply because of there being a larger proportion of such people in the sample population.

Importantly, none of the previously mentioned studies included family history as an independent criterion to initiate surveillance, as we suggested above, despite evidence that family history increases the incidence of HCC in HBV patients.^37^ No data from the risk score studies is available to calculate what percentage of the unsurveilled‐HCC would be additionally under surveillance if surveillance for family history of HCC was performed. From our data, using the risk scores the majority of patients with family history but without cirrhosis fell in the low‐risk category and would not have been surveilled. The proportion of HBV patients with a family history of HCC in a population can vary from 4.6^37^ to 16% in our study, depending on the population and the definition of family history.

Therefore, with loose extrapolation from the above data, and excluding family history as a factor, the percentage of HBV unsurveilled‐HCC falling outside the surveillance criteria in Thailand may be 10–14% of all HBV‐HCC using the traditional age–sex criteria, while for risk scores it may be 5–15% for REACH‐B, 14–65% for CU‐HCC, and 32–75% of GAG‐HCC. The corresponding reduction in the number of patients requiring ultrasound surveillance in the untreated and treated non‐cirrhotic HBV population would be 54.1 and 75.2% for REACH‐B, 58.8 and 75.2% for CU‐HCC, and 69.5 and 80% for GAG‐HCC, respectively. Some 5–16% increased surveillance and improvement in sensitivity may potentially be seen, with the addition of family history of HCC as an independent criterion for surveillance.

The implications from our study is that, if risk scores were used in non‐cirrhotic HBV patients, there would be a dramatic reduction in the number of patients needing HCC surveillance (by approximately 50–80%). This would reduce the resource burden of HCC surveillance immensely. Which HCC risk score should be used in Thailand needs to be assessed prospectively for its accuracy in HCC prediction in the Thai population. For cirrhotic patients, HCC risk scores appeared to be unnecessary due to the high risk of HCC development. Family history of HCC is not included as a risk factor in any HCC risk score calculations, and patients with a family history of HCC should probably have early screening until this risk factor is added to the risk scores.

In conclusion,

- In an HBV‐endemic country, using HCC risk scores in non‐cirrhotic HBV patients, with or without antiviral therapy, would reduce the number of patients needing surveillance dramatically, with the implication that there would be a corresponding reduction in the resources needed for HCC surveillance.
- HCC risk scores appeared to be unnecessary in HBV cirrhotic patients, and these patients should have surveillance without needing risk score calculation.
- As risk scores do no incorporate the family history of HCC into its risk calculation, non‐cirrhotic patients with a family history of HCC should undergo surveillance until it is included in the risk scores, or proven unnecessary in prospective trials.

## Appendix

**Table A1:** Summary hepatocellular carcinoma (HCC) risk scores used in untreated chronic hepatitis B (HBV) patients

HCC risk scores	REACH‐B^13^	CU‐HCC^15^	GAG‐HCC^14^	LSM‐HCC^16^
*n* (Cohort)	3584	1005	820	1035
*n* (validated)	1505	424	—	520
Place	Taiwan	Hong Kong	Hong Kong	Hong Kong
Race	Asian	Asian	Asian	Asian
Age (year)	45.7	48	40.6	46
HBeAg negative (%)	84.8	—	56.6	75
Cirrhosis (%)	0	38.1	15.1	32
F/U (year)	12	9.94	5.62	5.8
Antiviral therapy (%)	0	15.1 (during study)	0	38 (during study)
HCC (%)	131 (3.7)	105 (10.4%)	40 (4.4%)	38 (3.7%)
Parameters	Age, sex, HBV‐DNA, ALT	Age, Alb, HBV‐DNA, cirrhosis, TB	Age, Sex, HBV‐DNA, cirrhosis	Age, Alb, HBV‐DNA, LSM
Calculator	Male sex: 2 points Age: 1 point for every 5 years from 35 to 65 years of age (0–6 points) ALT (IU/L): 15–44 (1 point), ≥45 (2 points) Positive HBeAg: 2 points HBV DNA (log copies/mL): 4–5 (3 points), 5–6 (5 points), ≥6 (4 points)	14 × sex (male = 1; female = 0) + age (in years) + 3 × HBV DNA (log copies/mL) + 33 × cirrhosis (presence = 1; absence = 0)	Age (>50 years = 3; ≤50 = 0) + albumin (≤35 g/L = 20; >35 = 0) + bilirubin (>18 μmol/L = 1.5; <18 = 0) + HBV DNA (<4 log copies/mL = 0; 4–6 = 1; >6 = 4) + cirrhosis (yes = 15; no = 0)	Age (>50 years = 10; <50 = 0) + albumin (≤35 g/L = 1; >35 = 0) + HBV DNA (>200 000 IU/mL = 5; ≤200 000 = 0) + liver stiffness (≤8.0 kPa = 0; 8.1–12.0 = 8; >12.0 = 14)
*Predictive HCC incidence*				
Risk stratification from score	0–8: 0–0.8% 9–11: 1.2–3.3% ≥12–17: 5.3–47.4%	≥101: ~5%	<5: 0.9% 5–19: 5.5% ≥19: 21.2%	<11: 0.3–0.6% ≥11–30: 5.0–14.4%
AUROC for 5‐year HCC prediction	0.796	0.88	0.75	0.83

ALT, alanine aminotransferase; TB, tuberculosis.

**Table A2:** Summary of risk scores used in treated hepatitis B virus (HBV) patients

HCC risk scores	REACH‐B^13^	CU‐HCC^15^	GAG‐HCC^14^	mPAGE‐B^17^
*n* (Cohort)	3584	1005	820	2001
*n* (validated)	1505	424	—	1000
Place	Taiwan	Hong Kong	Hong Kong	Korea
Race	Asian	Asian	Asian	Asian
Age (year)	45.7	48	40.6	50
HBeAg negative (%)	84.8	—	56.6	65.8
Cirrhosis (%)	0	38.1	15.1	19.1
F/U (year)	12	9.94	5.62	4.1
Antiviral therapy (%)	0	15.1 (during study)	0	100
HCC (%)	131 (3.7)	105 (10.4%)	40 (4.4%)	132 (6.6%)
Parameters	Age, sex, HBV‐DNA, ALT	Age, Alb, HBV‐DNA, cirrhosis, TB	Age, Sex, HBV‐DNA, cirrhosis	Age, Sex, Platelet, Alb
Calculator	Male sex: 2 points Age: 1 point for every 5 years from 35 to 65 years of age (0–6 points) ALT (IU/L): 15–44 (1 point), ≥45 (2 points) Positive HBeAg: 2 points HBV DNA (log copies/mL): 4–5 (3 points), 5–6 (5 points), ≥6 (4 points)	14 × sex (male = 1; female = 0) + age (in years) + 3 × HBV DNA (log copies/mL) + 33 × cirrhosis (presence = 1; absence = 0)	Age (>50 years = 3; ≤50 = 0) + albumin (≤35 g/L = 20; >35 = 0) + bilirubin (>18 μmol/L = 1.5; <18 = 0) + HBV DNA (<4 log copies/mL = 0; 4–6 = 1; >6 = 4) + cirrhosis (yes = 15; no = 0)	Age (<30 years = 0, 30–39 = 3, 40–49 = 5, 50–59 = 7, 60–69 = 9, ≥70 = 11) + gender (female = 0, male = 2) + Platelets (×10^9^/L) (>250 = 0, 200–250 = 2, 150–200 = 3, 100–150 = 4, <100 = 5) + Albumin (g/dL) (≥4.0 = 0, 3.5–4.0 = 1, 3.0–3.5 = 2, <3.0 = 3)
*Predictive HCC incidence*				
Risk stratification from score	0–8: 0–0.8% 9–11: 1.2–3.3% ≥12–17: 5.3–47.4%	≥101: ~5%	<5: 0.9% 5–19: 5.5% ≥19: 21.2%	<9: 0.7% 9–12: 5.1% ≥13: 18.4%
AUROC for 5‐year HCC prediction	0.74	0.87	0.93	0.82

ALT, alanine aminotransferase; AUROC, area under the receiver operating characteristic curve; TB, tuberculosis.
